# Transition from traditional to modern forest management shaped the spatial extent of cattle pasturing in Białowieża Primeval Forest in the nineteenth and twentieth centuries

**DOI:** 10.1007/s13280-016-0795-4

**Published:** 2016-06-02

**Authors:** Tomasz Samojlik, Anastasia Fedotova, Dries P. J. Kuijper

**Affiliations:** 1 Mammal Research Institute, Polish Academy of Sciences, ul. Waszkiewicza 1c, 17-230 Białowieża, Poland; 2 Institute for the History of Science and Technology, Russian Academy of Sciences, Universitetskaya nab. 5, St. Petersburg, Russia 199034

**Keywords:** Environmental history, Forest management, Forest regeneration, Historical ecology, Livestock impact, Wood pasture

## Abstract

Pasturing of livestock in forests has had profound consequences for Europe’s landscapes. In Białowieża Primeval Forest (BPF), cattle pasturing was a part of traditional forest use that ceased only in the second half of the twentieth century. We collected information on the institutional changes governing forest cattle pasturing and the changes in spatial extent of cattle presence in BPF in last two centuries and information on cattle numbers and their impact on forest regeneration. The spatial extent of cattle pasturing was highly variable, with the distribution of grazing areas frequently changing. Forest near villages (constituting less than 10 % of the area) was most often used for cattle grazing during continued longer time periods. Historical data showed that cattle have had a clear impact on forest regeneration. However, the frequent changes that occurred in the extent of cattle grazing indicate that their impact occurred locally, was smaller in other less intensively used areas, and in the forest as a whole.

## Introduction

Traditional management of forest resources in pre-industrial times was mainly shaped by two driving forces: socio-economic factors (i.e. demand for resources) and ecological conditions (Szabó and Hédl 2013; Müllerová et al. 2014). It has led to the development of local, informal ways of forest management balancing those two factors in European lowland forests. Traditional types of forest resource use like coppicing, haymaking on forest meadows or wood pastures (Rackham 2004; Bergmeier et al. 2010) existed without or with very little formal institutional governance. After the modern forestry management was introduced in the eighteenth century and nineteenth century, the forest exploitation was put under the formal institutional governance. The development of formal institutions (e.g. state forestry, ministries of forests) and enforcement of their values (e.g. prioritizing timber production and financial revenue, reorganizing forest structures and simplifying their biological composition according to forest management plans, restricting access to the forest), changed management perspectives and contradicted the traditional forest management (Hölzl 2010). The informal ways of forest use were considered obsolete or even harmful for the “correct” woodland development and were gradually removed from both private- and state-owned forests. The shift towards formal governance over forest management disconnected the social-ecological systems from their resources at local scale and has led, in the longer term, to cultural severance and loss of traditional forest knowledge (Rotherham 2007).

One of the main ways of traditional forest use in Europe was pasturing of domestic animals (cattle, sheep, pigs, goats, horses), a practice as ancient as the domestication of these animals itself, reaching back to the Neolithic Period (Hamilton et al. 2009; Szabó 2013; Yalden 2013). As such, it has had profound consequences for natural habitats and was one of the main factors shaping European landscapes (Vera 2000). After opening the canopy by means of fires or cutting, cattle pasturing contributed to maintaining openness inside the forest (Ericsson et al. 2000), aided by other human-related disturbances. Anthropogenic activities (forest felling, burning, mowing) and livestock presence, in combination with natural factors, has been recognized as one of the main causes for the decline of deciduous forests in Sweden (Björse and Bradshaw 1998) and the Netherlands (Dirkx 1998), and for deforestation of Great Britain (Clutton-Brock 1989).

Long-term presence of livestock, together with human management of trees and shrubs across Europe led to creation of so-called “wood pasture” (Rackham 2013; Rotherham 2013). Nowadays, these landscapes are valued for their high biodiversity (Bokdam and Gleichman 2000; Frelechoux et al. 2007; Bergmeier et al. 2010; Hartel et al. 2013; Öllerer 2013). Wood pastures are a well-researched topic but livestock grazing inside forests is little known. It was proved to have a significant impact on the woodland structure (Buttenschon and Buttenschon 2013), plant species diversity (Humphrey and Patterson 2000) and animals associated with them (Mitchell and Kirby 1990). In general, livestock grazing negatively affects the regeneration of tree species if they are not fenced or protected by thorny shrubs (Mayer and Stöckli 2005; Mayer et al. 2005; Hjeljord et al. 2014). Combined with other traditional activities it can contribute to the development of heterogeneously structured forest stands (Mayer and Stöckli 2005; Mayer et al. 2005).

There are not many studies covering the institutional transitions (from informal to formal) governing woodland management in Europe and emphasizing changes from the traditional to modern forest management paradigms (e.g. Szabó and Hédl 2013), yet it seems such narratives can play a crucial role in understanding the historical anthropogenic impacts on woodlands. The majority of studies on impact of livestock pasturing on forests in Europe were conducted in areas which were intensively used and underwent significant anthropogenic modifications to the point when they were turned into semi-open wood pastures or deforested totally. Little is known on the role of livestock in shaping forest ecosystems that were always forested and meant to stay a closed forest. Only some historic information exists on the impact of cattle pasturing on forest regeneration, soil condition and botanical composition of pastured forest floor (Vysotskii 1926; Falkovskii 1928, 1929). Our study on Białowieża Primeval Forest (BPF) on the border of Poland and Belarus offers a unique glimpse into the history of livestock pasturing inside the forest. Here, this activity used to be common a century ago (Faliński 1966, 1986; Jędrzejewska et al. 1997), yet the area has always been dominated by forest. The general assumption was that cattle were a major destructive force in the forest (e.g. Paczoski 1930; Faliński 1986) but the details on the actual impact this former livestock grazing had are largely unknown. As a royal forest, BPF has been protected since the fourteenth century (Samojlik 2010), and has had a long-lasting tradition of multi-functional use, including pasturing of livestock (Hedemann 1939). The current knowledge on livestock presence in BPF only refers to scarce information on livestock numbers (Jędrzejewska et al. 1997; Samojlik and Kuijper 2013) but little information exists on the spatial extent of animals present in woodlands. Especially, their distribution is crucial to understand what impact they really had in this forest system which, in turn, might shed new light on similar processes in lowland temperate forests throughout Europe.

Next to the quantification of numbers of livestock present in BPF in the nineteenth and twentieth centuries (Jędrzejewska et al. 1997), there was only an attempt to map the distribution of areas with cattle pasturing in the twentieth century, but without further analysis of livestock impact (Faliński 1966, 1986). Our work will be the first combining the analysis of unpublished historical maps showing the actual spatial extent, and the variation between years, of pasturing areas in the BPF and the only known dataset showing the impact of historical cattle presence on forest regeneration on the background of institutional changes governing traditional use of forest. These will help to understand how important livestock pasturing was in shaping European lowland forests in the past.

The goal of our paper is to (1) reconstruct the main turning points in the transition from informal to formal institutional governance over management of livestock pasturing in BPF in the nineteenth and twentieth centuries; (2) assess the spatial extent of cattle pasturing inside BPF during the nineteenth and twentieth centuries; (3) assess the number of cattle present in BPF; (4) estimate the impact of cattle pasturing in the forest in the middle of the twentieth century. These insights in the extent and impact of historical livestock pasturing will be discussed in the light of modern forest management practices involving cattle in European lowland forests.

## Materials and methods

### Study area

**Fig. 1:**
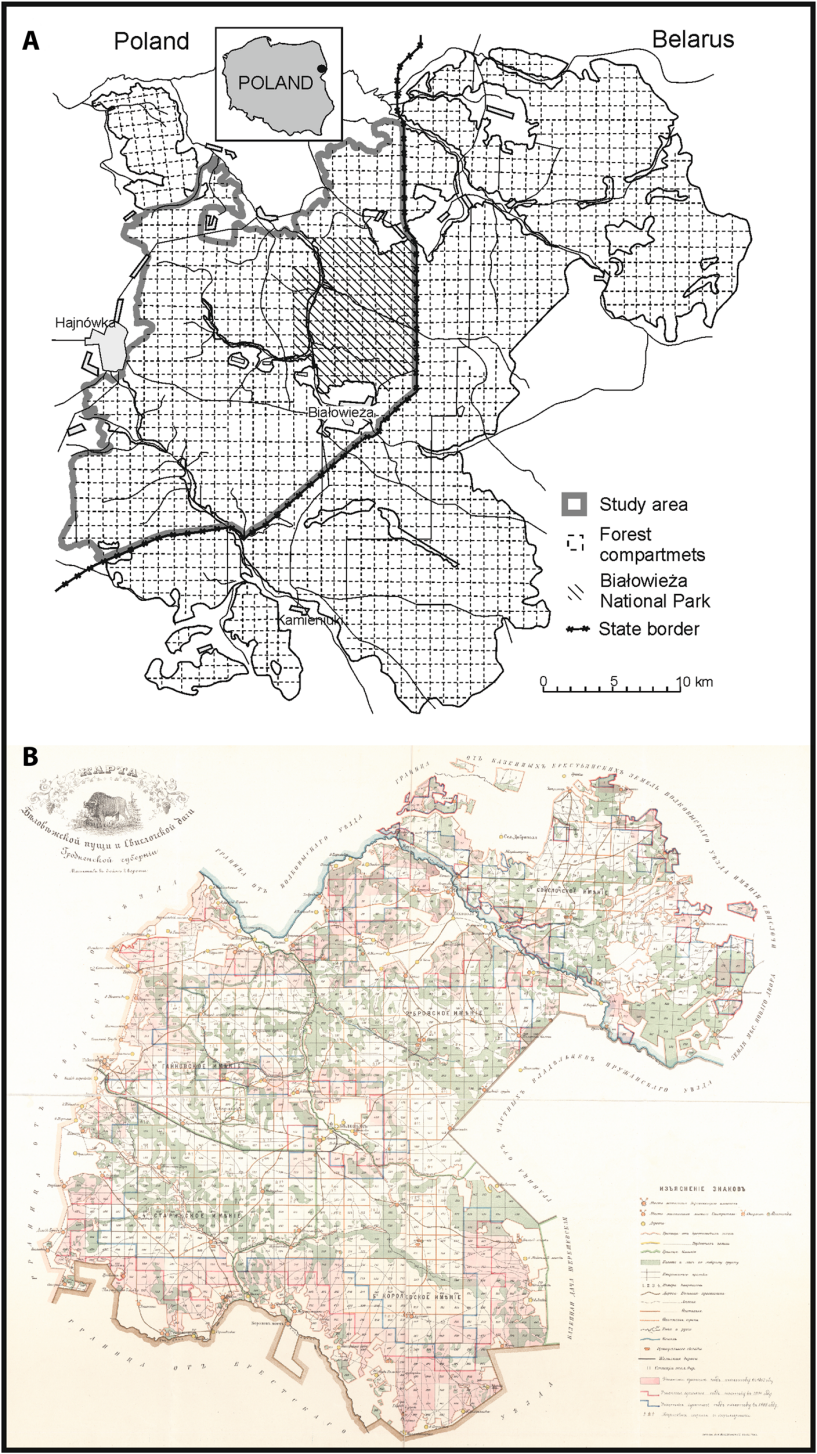
**a** *Map* of the contemporary Białowieża Primeval Forest (BPF, Polish and Belarusian part) with the study area marked (drawn by T. Samojlik). **b** The archival *map* showing the extent of pastures in the Polish and Belarusian parts of BPF in 1897 (in *pink*), 1904 (*red line*) and 1905 (*blue line*) (*Map* of Białowieża Forest 1905)

Białowieża Primeval Forest, covering 1450 km^2^, is a continuous temperate mixed lowland forest located on the border of Poland and Belarus, with 600 km^2^ on the Polish side (Fig. 1). The Polish part incorporates the Białowieża National Park of 105 km^2^ (with 47.5 km^2^ of old-growth forest strictly protected since 1921) with no human intervention allowed, and forests managed under state forestry. In the managed part of the forest, mass-scale timber exploitation with clear cuts and forest plantations was carried out since 1915, with a significant decrease in wood production since the end of the twentieth century. The managed part of the forest has generally younger age class-distribution of the tree stands than the strictly protected stands inside the national park (Jędrzejewska and Jędrzejewski 1998).

BPF is a rich, multispecies, closed canopy forest (in Białowieża National Park −99.2 % of the area; Michalczuk 2001) with a small share of open areas—river valleys, marsh lands and forest gaps. The forest consists of five main forest types: coniferous (with *Pinus sylvestris* and *Picea abies* dominating), mixed coniferous (*P. sylvestris, P. abies* and *Quercus robur*), deciduous (*Q. robur*, *Tilia cordata* and *Carpinus betulus*), mixed deciduous (*P. abies*, *Q. robur*, *T. cordata* and *C. betulus*) and wet black alder bog forest and streamside alder-ash forest (*Alnus glutinosa* and *Fraxinus excelsior*). In the Polish part of BPF, 52 % of the wooded area is covered by coniferous forest, and in Belarusian part coniferous stands straddle over 69 % of the area (Jędrzejewska and Jędrzejewski 1998).

The climate is transitional between Continental and Atlantic, with mean annual temperatures reaching 7.0 °C (−4.8 °C for January and 18.4 °C for June) and average annual precipitation of circa 640 mm (Jędrzejewska and Jędrzejewski 1998).

### Sources for historical range of cattle grazing in BPF

A survey in search for historical maps and documents on the management of BPF (especially the regulations concerning livestock pasturing) in the nineteenth and twentieth centuries was conducted in State Archive in Białystok, Regional Directorate of State Forests, Poland, and Russian State Historical Archive in St. Petersburg, Russia (RGIA). The survey yielded several previously unknown documents on the informal and formal policy regulations of pasturing practices in BPF, including two maps (showing four different time periods of pasturing in the BPF) and a manuscript of previously unpublished study of restocking success under cattle pressure (Reindl and Krukowski 1958). Additionally, a review of the literature concerning the management and wildlife of BPF published in the nineteenth and twentieth century (Genko 1902–1903; Karcov 1903, Kocan 1957; Więcko 1963, 1984; Faliński 1966, 1986; Jędrzejewska et al. 1997) was conducted, yielding another two maps showing the range of cattle pasturing and general information.

The analysis of changes in areal extent of cattle pasturing inside the forest in the nineteenth and twentieth centuries was based on series of four historical maps (one of which contains information on 3 different periods) with cattle pasturing areas indicated:

1. Map of Białowieża Forest and Świsłocz Forestry in Grodno Province, showing the pasturing range in 1897, 1904 and 1905 (Map of Białowieża Forest 1905; Fig. 1).
2. Map of BPF in 1902 showing the areas designated for game species in autumn and the compartments under pasture (Karcov 1903).
3. Map showing the area under cattle pasturing in the Polish part of BPF in 1956 (Reindl and Krukowski 1958).
4. Map showing the area under cattle pasturing in the Polish part of BPF in 1964 (Faliński 1966, 1986).

### Sources and calculations of cattle numbers

To reconstruct the numbers of cattle present in the area we used four different types of information derived from the above-mentioned sources: (1) direct numbers of cattle present in the forest (from the interwar and post-war period), (2) annual income from pasturing to the BPF administration (from 1883 to 1910), (3) annual calculations of area under pasture, given in desyatinas (1 desyatina = 0.0109275 km^2^) (1883–1893) and (4) number of cow-units, for which the charges to the BPF administration were paid in the period 1897–1909. In the latter case, calves and oxen were counted as half of a cow-unit, as both calves (young and small) and oxen (used for work and less often present on pastures) were using less resources than adult cows (Więcko 1984). If the sources contained data on annual income from pasturing and on area of the forest under pasture, we recalculated it directly to cattle numbers. For the period 1883–1893, cattle owners were obliged to pay for the area of forest pastures (1 rouble for 10 desyatinas) and were able to keep 1 cow for 5 desyatinas (on the change of cattle pasturing law in the forest since 2 September 1869). For the period since 1894, the payment no longer depended on the area of pasture but on the number of cow-units. Prices for the cow-unit were changing over time: in the end of nineteenth century (1894–1999) the price was 1.40 roubles for cow-unit, in 1900 it was lowered to 1.20 roubles and in 1905 further reduced to 1 rouble (on allowing different persons to pasture in Białowieża Forest for pay and free of charge 1908). Using this information, we were able to recalculate the annual pay and desyatinas under pasture into cattle numbers (1883–1893) and cow-units (1894–1910). Since the proportion of adult cows, calves and oxen in BPF villages in the nineteenth and beginning of the twentieth century is not known, we were not able to unify the above-mentioned units.

To calculate cattle numbers present in the study area (contemporary Polish part of BPF) for the years in which data are present for the entire BPF, we have used the data from 1909 document with a list of villages and number of cow-units (On Białowieża Forest. On allowing different persons to pasture cattle in forests for pay and free of charge 1909). Based on this, we have calculated that villages located on the contemporary Polish side of the border dividing BPF held 56 % of cow-units present in the entire BPF in 1909. Assuming that the proportion of cattle ownership of all villages did not differ much between years, we have used this factor for overall cattle numbers in years 1883–1910 (Table 1).

**Table 1:** The area with pasturing allowed and the number of cattle present in BPF in 1875–1969 based on archival survey and data from literature review. (*) Marks years for which cow-units, not actual cattle numbers, are given. (**) Indicates years for which cattle numbers were calculated on the basis of the annual income from pasturing (in Russian roubles) or the information on area grazed by cows (in desyatinas). Sources used (1) Faliński 1966; (2) Faliński 1986; (3) Jędrzejewska et al. 1997; (4) Karcov 1903; (5) Kocan 1957; (6) Plan urządzenia… 1958–1968; (7) Reindl and Krukowski 1958; (8) Report from Kolokoltsev to the Ministry of State Domains from 19 March 1906; (9) On exchange of state forest properties in Białowieża Forest and Świsłocz estate for appanage lands 1886–1895; (10) Map of Białowieża Forest 1905; (11) On Białowieża Forest. On allowing different persons to pasture cattle in forests for pay and free of charge 1909; (12) On the outbreak of anthrax and the fight with it 1910; (13) State Forestry Directorate 1931; (14) Więcko 1963; (15) Więcko 1984; (16) Wróblewski 1927

Year	Cattle numbers		Area under pasture (km^2^)		Source	Historical data on income/area
	Entire BPF	Study area	Entire BPF	Study area		
1875	–	–	457	–	3	**
1883	7568	4253	413	–	9	** 3 784 roubles
1884	7452	4188	407	–	9	** 3 726 roubles
1885	8230	4625	450	–	9	** 4 115 roubles
1886	8600	4833	470	–	9	** 4 300 roubles
1887	8412	4727	460	–	3	** 4 206 roubles
1888	6348	3568	467	–	14	** 4 273 roubles
1888	8546	4802		–	8	
1889	7330	4119	475	–	9	
1889	9886	5556	540		3, 14	** 4 943 roubles
1892	10400	5845	568	–	4	** 52 000 desyatinas
1894	–	–	399	–	11	** 36 586 desyatinas
1897	5947.5*	3342.5*	270	184.57	10	** 8 327 roubles; 24679 desyatinas
1898	–	–	286	–	4	** 2 6219 desyatinas
1900	4959.5*	2787*	286		3, 14	** 6 943 roubles
1901	5124*	2880*	–	–	14	** 6 149 roubles
1902	4916.5*	2763*	–	99.96	4, 14	** 5 909 roubles
1904	4533.5*	2548*	214	95.76	10	** 19 596 desyatinas
1905	9299*	5226*	441	189.88	10; 11	** 4 738 roubles; 40 389 desyatinas
1908	6823*	3834.5*	441	189.88	11; 16	
1909	5253.5*	2952.5*	277	–	11	** 25 328 desyatinas
1910	7500*	4215*			12	
1931	~5000	–	–	–	13	
1954	–	1750	–	–	6, 5	
1955	–	1947	–	–	5, 6	
1956	–	2197	–	151.14	6, 7	
1956	–	3136	–	–	5	
1957	–	3136	–	103.31	6, 15	
1958	–	3620	–		6	
1964	–	–	–	105.05	1, 2	
1966		1400			1	
1967	–	814	–	51.06	15	
1969	–	405	–	–	15	

### Reconstruction of formal and informal institutional development of livestock pasturing

Documents and literature gathered during the archival survey were analysed from the point of view of institutional bodies governing the traditional use of the forest. All information directly or indirectly referring to institutional settings governing woodland grazing were extracted, and the evolution of those settings was reconstructed.

### Historical impact of cattle grazing

**Fig. 2:**
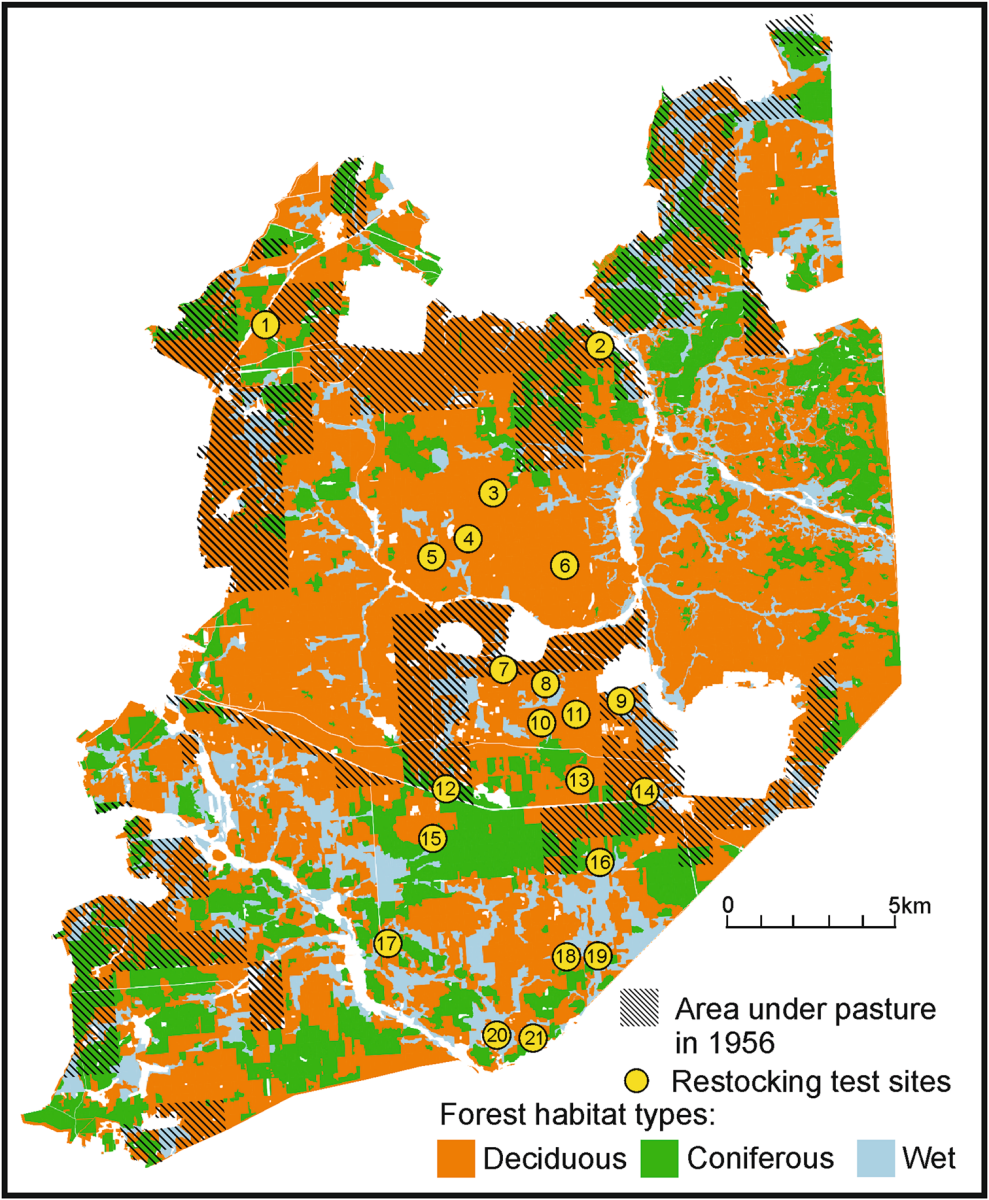
Plots used in the restocking success test from 1956 on the background of current vegetation map of the Polish part of Białowieża Primeval Forest, with the area under pasture in 1956 marked (drawn by T. Samojlik)

Data on the impact of cattle grazing on forest plantations were based on a study carried out in 1956 by the Regional Directorate of State Forests in Białystok (Reindl and Krukowski 1958), when 2197 cattle were pastured on 151.14 km^2^ of the forest (0.14 cattle/ha). In this study, the survival of seedlings (restocking success) was determined on 192 forest plantations on fresh clearcuts in 21 forest compartments located in different habitats (see Fig. 2). In each plantation, seedling survival was checked by walking planting rows and counting the surviving trees, with the results being shown as the percentage of survivors. Seedlings were planted according to the management plan for different forest habitat types, mainly Scots pine and oak with admixture of other species (Reindl and Krukowski 1958).

In the original seedling survival study, all 192 sites were divided in three groups: (1) sites with cattle present and located <5 km from foresters’ houses; (2) sites without cattle, >5 km from the foresters’ houses; (3) sites without cattle, closer than 5 km from foresters’ houses (Reindl and Krukowski 1958). The distance from the foresters’ houses is relevant because the closer locations had better control by forest service and therefore were better protected. At locations farther from foresters’ houses, the reduced control often led to illegal presence of cattle. Other factors that can potentially influence the seedling survival, like climatic conditions, presence of wildlife, technique of restocking, quality and methods of seedlings tending, were not taken into account (Reindl and Krukowski 1958).

This historic inventory was not designed in the first place as a scientific study, thus the distribution of 192 sites among three groups and among different forest habitats (and, consequently, the species planted in three groups of sites) was not equal. However, being the only written source available, we believe that this study gives a valuable estimation of the impact of cattle in historical times.

### GIS and statistical analyses

We used Quantum GIS (QGIS 2015) for creating maps and analysing changes in spatial extent of cattle grazing. In all these analyses, we selected only that area for which we had information on cattle grazing covered by all the historical maps. This area covered the contemporary Polish part of BPF without the north-western tip, Ladzka Forest, which was incorporated to BPF only after WWII (Więcko 1984). The entire area covered by analysis is circa 580 km^2^, see Fig. 1. A series of six maps was used to estimate the spatial extent and the changes in livestock grazing in the forest. To indicate which areas were most impacted by historical livestock grazing, we overlaid all historical maps. Afterwards, different parts of the forest were divided into three categories: areas with high (cattle present in 5 or 6 periods), medium (3 or 4 periods) and low (1 or 2 periods) pasturing frequency.

For analysis of statistical difference between three categories of sites in terms of seedling survival we used Kruskal–Wallis rank-sum test, and for testing the differences between pairs of categories—Wilcoxon rank-sum test. Calculations were conducted in R programme (R Core Team 2015).

## Results

### Informal and formal policies regulating forest grazing in BPF in the nineteenth and twentieth centuries

All sources show that only cattle were legally pastured in BPF, never mentioning other livestock as goats, sheep or pigs entering the forest. The only exception is the document describing attempts to receive compensation for peasants’ horses killed by bison which made clear that horses should not be pastured inside BPF (On issuing compensations for different persons for horses killed by bison 1898–1899).

#### Shift between the informal and formal governance of cattle forest pasturing in the period 1795–1888

After the third partition of Poland and seizing control over the entire BPF by Russian Empire, the majority of traditional forest uses continued. Cattle pasturing inside BPF, which was present here at least since the sixteenth century (Hedemann 1939; Samojlik 2007), was one of such uses. The area of forest under pasture was not strictly delimited and followed the eighteenth-century stipulations that cattle should be pastured in areas adjacent to villages (Samojlik and Kuijper 2013). This informal, traditional right to pasture was beyond control of the administration. The first attempt at formalizing and controlling forest grazing was the state-level legal regulations on pasturing in forests issued by Forestry Department of the Ministry of State Domains of Russian Empire in 1864. Pasture in forests was to be allowed under several conditions, including the strict necessity for such use (On the conditions under which cattle pasturing is allowed in forests 1864). The first conflicts between the formal, state-level valuation of woodland (timber) and local, informal level valuation (traditional use, including pasturing) emerged, but due to the shortage in personnel and actual need for pastures in woodlands the authorities turned a blind eye to this activity (On the current state of Białowieża Forest and plans for its future management 1861).

The period 1860–1870s can be seen as transitional between the informal and formal governance of cattle grazing—laws on pasturing were not fully reinforced due to the shortage of qualified personnel. In the 1880s, laws on pasturing were adjusted: peasants were supposed to pay for their cattle (previously pastured without any charge) and fence the most vulnerable parts of the forest (On persecution for wilful pasturing of cattle in forest plots 1875–1883).

#### The period 1889–1914

In 1889, BPF was taken over by tsars’ personal treasure (Chief Department of Appanages), and the forest became a hunting reserve. Forest grazing was seen as damaging for game wildlife. The new ownership and formal regulation regimes continued to disconnect local communities from the woodland employing measures to control forest pasturing: (1) strict delimitation of the area of forest pastures ascribed to each of the villages; (2) consistent control over the annual pay for area of pasture and cattle brought to the forest; (3) contracts signed by each of the villages, in which the area of pasture is stated and obligations of cattle owners are listed. These actions triggered several conflicts at local scale (On the request from peasants of Podbielskie Ogrodniki to maintain their right to pasture cattle in BPF 1889; On complaints of Pogorzelce community on oppression from BPF administration, on not allowing them to earn in the forest and to pasture cattle 1902).

In theory, actions of the forest and game administration were aiming at displacing peasants from the forest. Apart from the usual complaints against pasturing of livestock (destroyed young trees, disturbing the soil, fires, poaching), game management saw the cattle as competitors for resources used by game animals. In reality, it was not possible without a major conflict. Even if the local administration managed to limit the area under pasture in the forest 1 year, crop failure in subsequent year forced it to make concessions to peasants (On the request from peasants of Podbielskie Ogrodniki to maintain their right to pasture cattle in BPF 1889).

Although the impact of cattle on vegetation was not assessed in this period, Paczoski (1897) noted that cattle grazing resulted in the increased presence of light-demanding juniper in forests.

#### The period 1915–1945

Limited information is available from the period 1915–1945. In the interwar period, cattle pasturing was allowed both in private and state forests by edicts of the Ministry of Agriculture (Broda 1965). State forest policies saw this type of use as one of the main destructive factors in the forest plantations, yet it was considered as a necessary evil (State Forestry Directorate 1931). All logged areas and forest tillage were to be excluded from pasturing for 15–20 years until the regenerating trees “escape the muzzles”, and all pastures and cattle driving roads—fenced by cattle owners. These plans remained on paper (State Forestry Directorate 1931), except for core area of BPF that was proclaimed a nature reserve in 1921. This fragment of the forest was from that point on excluded from pasturing.

Paczoski (1930) noted many undergrowth disturbances connected with pasturing of cattle, observed forests surrounding the settlements overgrazed on large areas, described peasants issuing an application to the Polish parliament to let them pasture cattle in the newly created reserve (which later became Białowieża National Park).

#### The period 1945–1973

After the Second World War, cattle were still pastured in the forest, due to the persistent problem of scarcity of pastures (Faliński 1986). Observations of the negative impact on the forest continued: Kocan (1957) wrote that cattle pasturing was not supervised and the livestock wandered around in the entire forest causing damage by browsing on seedlings, trampling birds’ nests, fouling the forest with dung. Faliński (1966) saw cattle presence together with overabundant game animals as the main obstacle in artificial and natural forest regeneration. Gradually, cattle grazing was separated from the forestry (Table 1), and eventually livestock pasturing was prohibited in all Polish state forests, including BPF, in 1973 (Więcko 1984).

### Changes in spatial extent of livestock pasture in BPF in 1875–1964

Regarding only the total area grazed, the archival and published written sources contained information about the period 1875–1969: entire BPF for 1875–1909, and the study area for 1954–1967 (without specifying the spatial distribution of parts of the forest under pasture). The area under grazing was more or less constant (on average 441 km^2^) in the period 1875–1888, until the transition of BPF to the tsars’ personal treasure (Appanages). Since then, the pasturing area started changing rapidly (in 1889–1910 it oscillated between 568 km^2^ at maximum and 214 km^2^ at minimum, averaging in this period 372 km^2^; Table 1). The data for the period 1956–1967, covering Polish part of the BPF, are much scarcer and show the decline of pastures from 151 km^2^ in the beginning to 51 km^2^ at the end of the period, 6 years prior to the final ban on pasturing (Table 1).

**Fig. 3:**
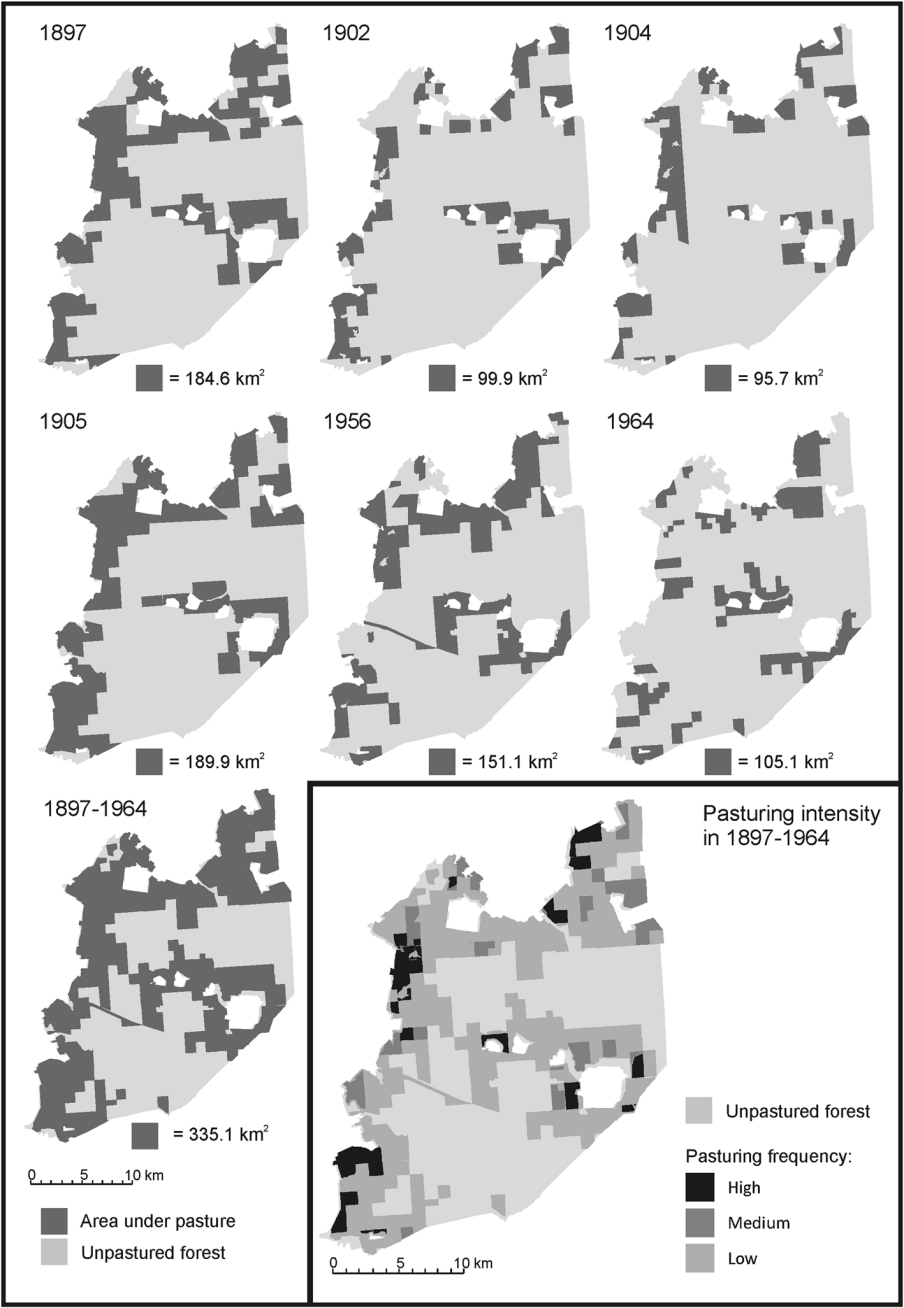
The area under pasture inside the study area (Polish part of BFP without Ladzka Forest) in six time periods, the cumulative area covered by this activity in the period 1897–1964 and the pasturing intensity in the above-mentioned period. Three levels of cattle grazing intensity were calculated: high—areas in which pasturing was present in 5 or 6 time periods, medium—areas with pasturing in 3–4 periods and low—areas in which pasturing occurred in 1 or 2 periods (drawn by T. Samojlik)

As regards the spatial distribution of the areas under pasture within BPF, collected maps contained information on six different periods: 1897, 1902, 1904, 1905, 1956 and 1964. Within the study area, the area used by livestock varied from 95.7 km^2^ in 1904 up to 189.9 km^2^ to 1905 (Fig. 3). The cumulative area used for grazing in the entire period 1897–1964 amounted to 335.1 km^2^, i.e. 58 % of the studied fragment of BPF. Maps from all six periods (Fig. 3) show that the majority of areas under cattle grazing were located in the forest adjacent to villages on the border and inside BPF, only occasionally in a distance larger than 2 km from the village. The chronological view at the spatial changes of pasturing area in the entire BPF and within the study site (including information from written sources in Table 1) shows that this area was not constant. As already evidenced in written historical sources, parts of the forest in which pasturing was allowed were changing between the years (see paragraph 3.1) (Fig. 3). The compilation of all maps showed that some areas have always been included in cattle pasturing, whereas others were only included in some periods. Parts of the area which had a high frequency of cattle grazing during the studied period covered 9.7 %, medium 10.3 % and low 80 % of the total area under pasture (335.1 km^2^). In other words, the majority of the forest under pasture was used by cattle only in one or two periods covered by the maps, and less than 10 % was constantly used for this purpose.

### The number of cattle present in BF

Cattle were brought to BPF for approximately 6 months per year, for several hours per day (from dawn till dusk). In the period 1883–1892, the number of cattle present in the entire BPF ranged from 8230 (or 6348 stated by previous studies, see Table 1) to 10 400, on average around 8000. This number seems well grounded in the historical information. In 1869, foresters stated that an average household is keeping 5 heads of cattle (On the change of cattle pasturing law in the forest since 2nd September 1869), whereas Genko (1902–1903) mentions that in 1884–1886 there were 1750 households from which cattle were brought to BPF (which gives an average of 4.6 animals per household).

For the period 1897–1910, only cow-units are available. In 1897–1904, the number was relatively stable (averaging circa 5000), yet in 1905 it rose to 9229; in the same year, the area under pasture was extended to the same degree (Fig. 3).

In the post-war period, numbers of cattle present in the Polish part of BPF show first an increase in 1954–1958 (from 1750 to 3620) and then a rapid decline to 405 animals in 1969 (Table 1).

### Historical impact of cattle grazing

**Fig. 4:**
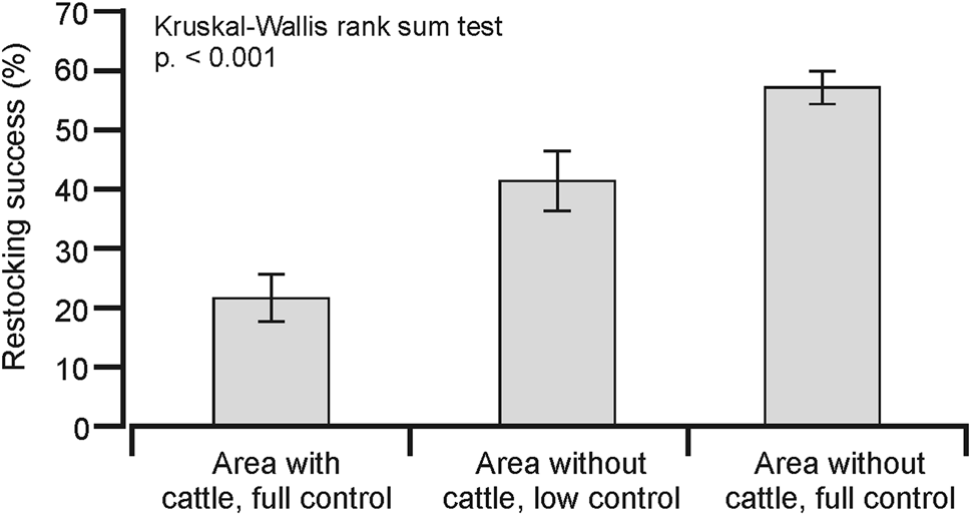
The restocking success tested in BPF in 1956 in three types of plots: (1) sites on areas with cattle present and located closer than 5 km from foresters’ houses (short: area with cattle, full control); (2) sites without cattle, but located over 5 km from the foresters’ houses, with lower control from forest service (area without cattle, low control); (3) sites without cattle, closer than 5 km from foresters’ houses (area without cattle, full control). Drawn by T. Samojlik

In areas with cattle and with close control, the average restocking success amounted to 21 % (±4 % SE), in areas without cattle and with low control to 41 % (±5 % SE), and in areas without pasturing and with close control to 57 % (±3 % SE) (Fig. 4). Significant differences between the seedling survival in three types of plots were found (Kruskal–Wallis *χ* ^2^ = 31.387, *df* = 2, *P* < 0.001), whereas the Wilcoxon rank-sum test between pairs of area types showed a strongly significant difference (*W* = 755.5, *P* < 0.001) between the 1st and 3rd area.

### Summary of the results

Our study showed that:

1. In the nineteenth and second half of the twentieth century, a transition from a local and informal to a state-based, formal regulation of forest cattle grazing occurred. This transition was paved with several conflicts and resulted in the disconnection of local people and traditional forest use (cattle pasturing) from their forest. Since 1945, the gradual decline of pasturing is observed until its eventual prohibition in 1973.
2. The spatial extent of cattle pasturing inside BPF during the nineteenth and twentieth centuries proved to be highly variable, with the distribution of grazing areas frequently changing. Only a small fraction (less than 10 %) of the study area was frequently (and likely for longer periods) used for cattle grazing, with the majority of forest used with medium and low frequency.
3. The number of cattle present inside BPF was constantly changing, most probably reflecting the administrative changes in pasturing area and the transition towards more formal, institutional governance over forest grazing.
4. The historical study from 1956 showed that livestock presence significantly reduced tree regeneration.

## Discussion

### Spatial extent of historical cattle grazing and cattle impact on forest

Livestock presence was argued to be one of the main factors shaping the forest landscapes of BPF in the nineteenth and twentieth centuries. Faliński (1966, 1986) saw it as a fundamental obstacle in the natural and artificial forest regeneration and as a source of ruderalisation of woods of BPF. It was even strengthened by the calculations showing that in the beginning of the twentieth century cattle composed up to 70 % of biomass of all ungulates (wild and domestic) present in the forest (Jędrzejewska et al. 1997). The impediment of forest regeneration by livestock is also confirmed by written sources and the unique 1956 survey, both presented here. Therefore, our study does not contradict the previous views on the impact of cattle, but rather puts it in a different perspective. Cattle had a negative effect on tree regeneration at local levels. However, only 10 % of the area had a high frequency of cattle grazing. Due to a large spatio-temporal variation in the grazed woodland surfaces, together with the variation in cattle number, cattle grazing had likely small impact on the forest as a whole. This variation, observed in our study, could be the key for sustainable woodland grazing strategy.

The variation occurred between all analysed periods, but was most striking between 1897 and 1905, when changes in pasturing area and cattle numbers were rapid and drastic. This can be explained by the development of the institutional governance of woodland grazing. In this period, the formal policy regulating woodland was for the first time firmly set and maintained by various means, including changes of pasturing areas often seen as a penalty for poaching and other misconducts. It is also possible that there was another reason for those changes: the depletion of resources. We do not have any knowledge on long periods in the history of forest grazing in BPF—it is probable that for several decades in the beginning of the nineteenth century the pasturing areas remained constant. Yet taking into consideration Paczoski’s (1930) and Faliński’s (1966, 1986) remarks on the destruction of undergrowth by cattle, it is possible that in the period before the formal, institutional toughened governance emerged, imposing its own regulations on pasturing area, this area could have been changed by cattle owners themselves just to avoid resource depletion.

### Limitations of historical data

Our spatial and numerical data on the area of pastures within BPF and numbers of cattle present, as well as information on restocking success, are fragmented and unevenly spread in time; nevertheless, we believe they give us a unique glimpse into the history of cattle pasturing and its possible implications for the forest. As for the restocking success, this is one of a kind study from the period when cattle were still present in BPF. Today, we can only plan to measure the long-lasting effects of past cattle presence in the forest.

The archival and historical data impose specific limitations on the study, e.g. presenting two different sets of units, actual number of animals and cow-units, which are not easily comparable. Furthermore, although we did our best to find historical sources for the entire study period, there are still gaps in the source material. The biggest, between 1910–1931 and 1931–1954, will be very difficult to cover by source materials, as most of the archives concerning the management of BPF were probably destroyed during the WWII. Nevertheless, there is always a possibility that new information will be discovered and our dataset will be updated and expanded, using the calculation methods between data on annual pay and area covered by pasturing to cattle numbers or cow-units proposed in this paper.

Our attempts at assessing the significance of cattle presence in BPF were based on collected maps and the calculation of the frequency with which certain areas were used as pasture. It is important to stress that three categories in map (Fig. 3) show only which areas were used most often, and it is not an indicator of an intensity of impact in ecological meaning. Nevertheless, we believe it is a good starting point to discuss the possible chronological and spatial disperse of the cattle impact, which is not immediately apparent when taking into account only bare numbers of animals and area used by them in a certain time point. We also believe that this is a first step in our quest for understanding the ecological meaning of cattle presence in the best preserved European lowland forest.

### Context of ecological research in BPF and Europe and practical implications

Results of this paper suggest that the historical ecology studies as this one could bring a valuable input to the ongoing discussion on the main factors shaping European forests in the past (Nilsson 1997; Bradshaw et al. 2003; Bradshaw 2004; Birks 2005; Mitchell 2005). This discussion includes also BPF as an example of a forest that was previously strongly influenced by livestock which, as some authors believe, maintained a mosaic landscape of grasslands with shrubs and small forested patches (Vera 2000). Although this approach was already criticized (e.g. Bobiec 2002) and is not supported by modern palynological analyses (Latałowa et al. 2015), this logic seems understandable in the light of information that in some years the total number of wild ungulates recorded in BPF was lower than the number of cattle pastured in the forest (e.g. in 1894 the total number of European bison, moose, red deer, fallow deer, roe deer and wild boar was estimated at 3040, with over 10 000 cattle present in the forest just 2 years prior; Karcov 1903). The disproportion is even more dramatic when looking at the biomass of domestic and wild ungulates, as mentioned above (Jędrzejewska et al. 1997). Nevertheless, as our study shows, cattle—contrary to wild animals—were kept on restricted areas and brought to BPF for limited time only. The area covered by cattle grazing, and especially the time of this pressure was then, respectively, smaller than the sheer number would suggest. We argue that our results show that a great amount of caution is needed before drawing far-reaching conclusions based only on cattle numbers or pasturing areas. The spatial and temporal analysis of historical data shows that the influence of cattle should be ascribed to a selected part of BPF only. The majority of the forest was devoid of livestock, and was rather influenced by changes in the wildlife management in the forest (Bobiec et al. 2011; Bobiec 2012; Kuijper et al. 2010), fire regime followed by its prohibition (Niklasson et al. 2010; Bobiec and Bobiec 2012), traditional uses of the forest resources (Samojlik 2005, 2006, 2007, 2009, 2010; Samojlik et al. 2013a, b, c) and, especially in the twentieth century, modern forest management.

## Conclusions

Currently, livestock pasturing is employed as a tool for nature management (Agnoletti et al. 2008; Hampicke and Plachter 2010), especially in attempts at re-wilding European woodlands or restoration of past wood pastures in European forests (e.g. Sumsion and Pollock 2005; Hodder and Bullock 2009; Buttenschon and Buttenschon 2013). We believe that such practices, mimicking the long-gone types of traditional use of forest ecosystems can serve their purpose very well. The management plans for wood pastures are obviously prepared with the historical knowledge as the main decisive factor (Rotherham et al. 2008; Perry 2013), yet it seems that the need for a detailed environmental history study, especially in the light of accelerating loss of traditional knowledge (Rotherham 2007) is indispensable. In case of our study area, BPF, the pre-existing knowledge led to the general assumption that cattle had an enormous impact on the entire forest. Our study proves that any management decisions based on this picture would be wrong, as this kind of impact most probably influenced only a small portion of the forest; furthermore, the pressure was constantly shifting spatially and the number of cattle was fluctuating. In some areas, a detailed historical study could reveal similar varied pattern of livestock impact connected with shifts in policies regulating woodland grazing. Our detailed historical study suggests also that not livestock, but rather other anthropogenic factors, such as fire and changes in ungulate management (Kuijper et al. 2010; Niklasson et al. 2010; Bobiec 2012; Bobiec and Bobiec 2012) are main factors responsible for forest development in this area.

## References

[CR1] Agnoletti M, Anderson A, Johann E, Kulvik M, Saratsi E, Kushlin A, Mayer P, Montiel C (2008). The introduction of historical and cultural values in the sustainable management of European forests. Global Environment.

[CR2] Bergmeier E, Petermann J, Schröder E (2010). Geobotanical survey of wood-pasture habitats in Europe: Diversity, threats and conservation. Biodiversity Conservation.

[CR3] Birks HJB (2005). Mind the gap: How open were European primeval forests?. Trends in Ecology & Evolution.

[CR4] Björse G, Bradshaw R (1998). 2000 years of forest dynamics in southern Sweden: Suggestions for forest management. Forest Ecology and Management.

[CR5] Bobiec A (2002). “Grazing ecology” from the Białowieża Primeval Forest perspective. Acta Theriologica.

[CR6] Bobiec A (2012). Białowieża Primeval Forest as a remnant of culturally modified ancient forest. European Journal of Forest Research.

[CR7] Bobiec A, Kuijper DPJ, Niklasson M, Romankiewicz A, Solecka K (2011). Oak (*Quercus robur* L.) regeneration in early successional woodlands grazed by wild ungulates in the absence of livestock. Forest Ecology and Management.

[CR8] Bobiec A, Bobiec M (2012). Influence of spruce decline in stands of the Białowieża National Park on natural oak regeneration. Sylwan.

[CR9] Bokdam J, Gleichman JM (2000). Effects of grazing by free-ranging cattle on vegetation dynamics in a continental north-west European heathland. Journal of Applied Ecology.

[CR10] Bradshaw RHW (2004). Past anthropogenic influence on European forests and some possible genetic consequences. Forest Ecology and Management.

[CR11] Bradshaw RHW, Hannon GE, Lister AM (2003). A long-term perspective on ungulate–vegetation interactions. Forest Ecology and Management.

[CR12] Broda J (1965). Dzieje lasów, leśnictwa i drzewnictwa w Polsce.

[CR13] Buttenschon RM, Buttenschon J, Rotherham I (2013). Woodland grazing with cattle. Results from 25 ears of grazing in acidophilus pedunculate oak (*Quercus robur*) woodland. Trees, forested landscapes and grazing animals. A European perspective on woodlands and grazed treescapes.

[CR14] Clutton-Brock J (1989). Five thousand years of livestock in Britain. Biological Journal of the Linnean Society.

[CR15] Dirkx GHP, Kirby KJ, Watkins C (1998). Wood-pasture in Dutch common woodlands and the deforestation of the Dutch landscape. The ecological history of European forests.

[CR16] Ericsson S, Östlund L, Axelsson AL (2000). A forest of grazing and logging: Deforestation and reforestation of a boreal landscape in central Sweden. New Forests.

[CR17] Faliński JB (1966). Antropogeniczna roślinność Puszczy Białowieskiej jako wynik synantropizacji naturalnego kompleksu leśnego [Anthropogenic vegetation of Białowieża Primeval Forest as a result of synanthropization of the forest].

[CR18] Faliński JB (1986). Vegetation dynamics in temperate lowland primeval forests.

[CR19] Falkovskii PK (1928). Study on the impact of cattle pasturing on physical features of oak forest soil in Trostianitsa forestry. Trudy po Lesnomu Opytnomu Delu Ukrainy.

[CR20] Falkovskii PK (1929). Study on the impact of cattle pasturing on oak forest growth and productivity in Trostianitsa forestry. Trudy po Lesnomu Opytnomu Delu Ukrainy.

[CR21] Frelechoux F, Meisser M, Gillet F (2007). Secondary succession and loss in plant diversity following a grazing decrease in a wooded pasture of the central Swiss Alps. Botanica Helvetica.

[CR22] Genko, N. 1902–1903. Kharakteristika Belovezhskoi Pushchi i istoricheskiya o nei dannyya. Lesnoi Zhurnal 21: 1014–1056, 22: 1269–1302, 23: 22–56. (in Russian).

[CR23] Hamilton J, Hedges REM, Robinson M (2009). Rooting for pigfruit: Pig feeding in Neolithic and Iron Age Britain compared. Antiquity.

[CR24] Hampicke U, Plachter H, Hampicke U, Plachter H (2010). Livestock grazing and nature conservation objectives in Europe. Large-scale livestock grazing. A management tool for nature conservation.

[CR25] Hartel T, Dorresteijn I, Klein C, Mathe O, Moga CI, Ollerer K, Roellig M, von Wehrden H (2013). Wood-pastures in a traditional rural region of Eastern Europe: Characteristics, management and status. Biological Conservation.

[CR26] Hedemann, O. 1939. *L’histoire de la foret de Białowieża (jusqu’a 1798)*. Warsaw: Instytut Badawczy Lasów Państwowych, Rozprawy i Sprawozdania Seria A, Nr 1. (in Polish with French summary).

[CR27] Hjeljord O, Histol T, Wam HK (2014). Forest pasturing of livestock in Norway: Effects on spruce regeneration. Journal of Forestry Research.

[CR28] Hodder KH, Bullock JM (2009). Really wild? Naturalistic grazing in modern landscapes. British Wildlife.

[CR29] Holzl R (2010). Historicizing sustainability: German scientific forestry in the eighteenth and nineteenth centuries. Science as Culture.

[CR30] Humphrey JW, Patterson GS (2000). Effects of late summer cattle grazing on the diversity of riparian pasture vegetation in an upland conifer forest. Journal of Applied Ecology.

[CR31] Jędrzejewska B, Jędrzejewski W (1998). Predation in vertebrate communities. The Białowieża Primeval Forest as a case study.

[CR32] Jędrzejewska B, Jędrzejewski W, Bunevich AN, Miłkowski L, Krasiński ZA (1997). Factors shaping population densities and increase rates of ungulates in Białowieża Primeval Forest (Poland and Belarus) in the 19th and 20th century. Acta Theriologica.

[CR33] Karcov, G. 1903. *Belovezhskaya Pushcha. Eya istoricheskii ocherk, sovremennoe okhotniche khozaistvo i Vysochaishie okhoty v Puchche*. St. Petersburg: A. Marks. (in Russian).

[CR34] Kocan, T. 1957. Szkodliwe wypasy w Puszczy Białowieskiej. *Przyroda Polska R.1, nr* 3: 6–7. (in Polish).

[CR35] Kuijper DPJ, Jędrzejewska B, Brzeziecki B, Churski M, Jędrzejewski W, Żybura H (2010). Fluctuating ungulate density shapes tree recruitment in natural stands of the Białowieża Primeval forest, Poland. Journal of Vegetation Science.

[CR36] Latałowa M, Zimny M, Jędrzejewska B, Samojlik T, Kirby KJ, Watkins C (2015). Białowieża Primeval Forest: A 2000-year interplay of environmental and cultural forces in Europe’s best preserved temperate woodland. Europe’s changing woods and forests: from wildwood to cultural landscapes.

[CR37] Map of Białowieża Forest and Świsłocz Forestry in Grodno Province. (1905) [Karta Belovezhskoi Pushchi i Svislotskoi Dachi Grodnenskoi Gubernii 1905]. RGIA, F. 515, Op. 42, No. 4572. (in Russian).

[CR38] Mayer AC, Estermann BL, Stöckli V, Kreuzer M (2005). Experimental determination of the effects of cattle stocking density and grazing period on forest regeneration on a subalpine wood pasture. Animal Research.

[CR39] Mayer AC, Stöckli V (2005). Long-term impact of cattle grazing on subalpine forest development and efficiency of snow avalanche protection. Arctic, Antarctic, and Alpine Research.

[CR40] Michalczuk C (2001). Siedliska i drzewostany Białowieskiego Parku Narodowego. Phytocenosis Supplementum Cartographiae Geobotanicae.

[CR41] Mitchell FJG (2005). How open were European forests? Hypothesis testing using palaeoecological data. Journal of Ecology.

[CR42] Mitchell FJG, Kirby KJ (1990). The impact of large herbivores on the conservation of semi-natural woods in the British uplands. Forestry.

[CR43] Müllerová J, Szabó P, Hédl R (2014). The rise and fall of traditional forest management in southern Moravia: A history of the past 700 years. Forest Ecology and Management.

[CR44] Niklasson M, Zin E, Zielonka T, Feijen M, Korczyk AF, Churski M, Samojlik T, Jędrzejewska B (2010). A 350-year tree-ring fire record from Białowieża Primeval Forest, Poland: Implications for Central European lowland fire history. Journal of Ecology.

[CR45] Nilsson SG (1997). Forests in the temperate-boreal transition: Natural and man-made features. Ecological Bulletins.

[CR46] On allowing different persons to pasture in Białowieża Forest for pay and free of charge. 1908. [O razreshenii raznym litsam prava pastby v lesnyh dachah BP za platu i bezplatno, 1908]. RGIA, F. 515, Op. 43, No. 825. (in Russian).

[CR47] On Białowieża Forest. On allowing different persons to pasture cattle in forests for pay and free of charge. 1909. [Po Belovezhskoi Pushche. O razreshenii raznym litsam prava pastby skota v lesah besplatno i za platu, 1909]. RGIA, F. 515, Op. 80, No. 648 (in Russian).

[CR48] On complaints of Pogorzelce community on oppression from BPF administration, on not allowing them to earn in the forest and to pasture cattle. 1902. [Po zhalobe krestian Pohorelskoho obshchestva na pritesnienia so storony administratsii BP, o nedopushchenii ih k zarabotkam v Pushche i k pastbe skota, 1902]. RGIA, F. 515, Op. 43, No. 442. (in Russian).

[CR49] On exchange of state forest properties in Białowieża Forest and Świsłocz estate for apanage lands. 1886–1895. [Ob obmienie kazennykh lesnykh dach Belovezhskoi Pushchi i Svislotskoi dachi na udelnyie zemli 1886–1895]. RGIA, F. 515, Op. 42, No 3415. (in Russian).

[CR50] On issuing compensations for different persons for horses killed by bison. 1898–1899. [O vydache raznym litsam posobii za ubityh zubrami loshadei 1898–1899]. RGIA, F. 515, Op. 43, No 283, 284. (in Russian).

[CR51] On persecution for wilful pasturing of cattle in forest plots. 1875–1883. [O presledovanii za samovolnuiu pastbu skota v lesnykh dachakh 1875–1883]. RGIA, F. 387, Op. 3, No 27825. (in Russian).

[CR52] On the change of cattle pasturing law in the forest since 2nd September. 1869. [Ob izmienienii pravil pastby skota v lesah. Zhurnal Spetsialnoho po lesnoi chasti komiteta ot 2 sent. 1869]. RGIA, F. 387. Op. 25. D. 52. pp. 42–80. (in Russian).

[CR53] On the conditions under which cattle pasturing is allowed in forests. 1864. [Ob usloviah pri kotoryh vozmozhna pastba skota v lesah 1864]. RGIA, F. 387, Op. 37, No. 638, pp. 114–137. (in Russian).

[CR54] On the current state of Białowieża Forest and plans for its future management. 1861. [O nastoiashchem sostoianii BP i o predpolozheniah o budushchem ee ustroistve. Zhurnal Spetsialnogho do lesnoi chasti komiteta 1861]. RGIA, F. 387, Op. 26, No. 27. (in Russian).

[CR55] On the outbreak of anthrax and the fight with it. 1910. [O poiavleniuu sibirskoi iazvy i borbe s niei 1910]. RGIA, F. 515, Op. 80, No. 775 (in Russian).

[CR56] On the request from peasants of Podbielskie Ogrodniki to maintain their right to pasture cattle in BPF. 1889. [Po prosbe ot upolnomochennoho ot krestian Podbielskie Ogorodniki o predostavlenii prava pastby skota v BP 1889]. RGIA, F. 515, Op. 43, No. 38. (in Russian).

[CR57] Öllerer K (2013). The vegetation of the Breite wood-pasture (Sighişoara, Romania)—History, current status and prospects. Brukenthal. Acta Musei, VIII.

[CR58] Paczoski J (1897). Flora Poles’ja i prilezhashhih mestnostej. Trudy Sankt-Petersburskhogo Obshchestva Estestvoispytatelei.

[CR59] Paczoski, J. 1930. *Lasy Białowieży*, 1–575. Poznań: Państwowa Rada Ochrony Przyrody. (in Polish with German summary).

[CR60] Perry S, Rotherham ID (2013). A strategic view of the issues for wood-pasture and parkland conservation in England. Trees, forested landscapes and grazing animals. A European perspective on woodlands and grazed treescapes.

[CR61] Plan urządzenia gospodarstwa leśnego nadleśnictw Puszczy Białowieskiej. 1958–1968. Tom I—Elaborat—ogólne opisanie [Forest management plan of forest districts in Białowieża Forest 1958–1968. Part I—General description]. Regional Directorate of State Forests in Białystok (unpublished manuscript in Polish).

[CR62] QGIS Development Team. 2015. Quantum GIS Geographic Information System. Open Source Geospatial Foundation Project. (http://qgis.osgeo.org).

[CR63] R Core Team. 2015. *R: A language and environment for statistical computing*. Vienna: R Foundation for Statistical Computing. (www.R-project.org).

[CR64] Rackham O (2004). Trees & woodland in the British landscape. The compete history of Britain’s trees, woods & hedgerows.

[CR65] Rackham O, Rotherham I (2013). Woodland and wood-pasture. Trees, forested landscapes and grazing animals. A European perspective on woodlands and grazed treescapes.

[CR66] Reindl, A., and S. Krukowski. 1958. Przegląd metod zagospodarowania lasów Puszczy Białowieskiej stosowanych w okresie lat 1918–1956 [The review of the methods of forest management employed in Białowieża Forest in 1918–1956]. Regional Directorate of State Forests in Białystok (unpublished manuscript in Polish).

[CR67] Report from Kolokoltsev to the Ministry of State Domains from 19 March. 1906. [Raport Kolokoltseva v GUU ot 19 iulia 1906]. RGIA, F. 515, O. 80, No. 409, p. 26–30. (in Russian).

[CR68] Rotherham ID (2007). The implications of perceptions and cultural knowledge loss for the management of wooded landscapes: A UK case-study. Forest Ecology and Management.

[CR69] Rotherham ID, Rotherham ID (2013). Grazed trees and landscapes. Trees, forested landscapes and grazing animals. A European perspective on woodlands and grazed treescapes.

[CR70] Rotherham ID, Jones M, Smith L, Handley C (2008). The woodland heritage manual: A guide to investigating wooded landscapes.

[CR71] Samojlik T (2005). A tree of many uses—The history of small-leaved lime (*Tilia cordata*) in Białowieża Primeval Forest. Rocznik Dendrologiczny.

[CR72] Samojlik T (2006). The grandest tree—A history of Scots pine (*Pinus sylvestris* L.) in Białowieża Primeval Forest until the end of the 18th century. Rocznik Dendrologiczny.

[CR73] Samojlik, T. 2007. Anthropogenic changes of the environment of Białowieża Primeval Forest until the end of 18th century. Ph.D thesis, Mammal Research Institute PAS, Białowieża. (in Polish).

[CR74] Samojlik T (2009). Bog iron ore extraction sites in the Białowieża Primeval Forest in the 17th–18th centuries. Kwartalnik Kultury Materialnej.

[CR75] Samojlik T (2010). Traditional utilisation of Białowieża Primeval Forest (Poland) in the 15th–18th centuries. Landscape Archaeology and Ecology.

[CR76] Samojlik T, Kuijper DPJ, Rotherham ID (2013). Grazed wood pasture versus browsed high forests: Impact of ungulates on forest landscapes from the perspective of the Białowieża Primeval Forest. Trees, forested landscapes and grazing animals. A European perspective on woodlands and grazed treescapes.

[CR77] Samojlik T, Rotherham ID, Jędrzejewska B (2013). Quantifying historic human impacts on forest environments: A case study in Białowieża Forest, Poland. Environmental History.

[CR78] Samojlik T, Jędrzejewska B, Michniewicz M, Krasnodębski D, Dulinicz M, Olczak H, Karczewski A, Rotherham I (2013). Tree species used for low-intensity production of charcoal and wood-tar in the 18th-century Białowieża Primeval Forest, Poland. Phytocoenologia.

[CR79] Samojlik T, Rotherham ID, Jędrzejewska B, Rotherham ID (2013). The cultural landscape of royal hunting gardens from the fifteenth to the eighteenth century in Białowieża Primeval Forest. Cultural severance and the environment.

[CR80] State Forestry Direcorate in Białowieża. 1931. Plan gospodarczy nadleśnictwa Hajnówka na 10-lecie 1931/32-1940/41 [The management plan of Hajnówka Forest District for the decade 1931/32-1940/41]. Regional Directorate of State Forests in Białystok. (unpublished manuscript in Polish).

[CR81] Sumsion, L., and M. Pollock. 2005. Woodland grazing toolkit. West Highland Woodland Grazing Project. Argyll and Bute Local Biodiversity Partnership.

[CR82] Szabó P, Rotherham I (2013). Rethinking pannage. Historical interactions between oak and swine. Trees, forested landscapes and grazing animals. A European perspective on woodlands and grazed treescapes.

[CR83] Szabó P, Hédl R (2013). Socio-economic demands, ecological conditions and the power of tradition: Past woodland management decisions in a Central European landscape. Landscape Research.

[CR84] Vera FWM (2000). Grazing ecology and forest history.

[CR85] Vysotskii GN (1926). On establishment of experimental forestry in Ukraine. Trudy po Lesnomu Opytnomu Delu Ukrainy.

[CR86] Więcko E (1963). The Białowieża Forest in 1795–1918. Kwartalnik Historii Kultury Materialnej.

[CR87] Więcko, E. 1984. *Puszcza Białowieska*, 1–309. Warszawa: Państwowe Wydawnictwo Naukowe. (in Polish).

[CR88] Wróblewski, K. 1927. *Żubr Puszczy Białowieskiej*, 1–232. Poznań: Wydawnictwo Polskie. (in Polish).

[CR89] Yalden D, Rotherham I (2013). The post-glacial history of grazing animals in Europe. Trees, forested landscapes and grazing animals. A European perspective on woodlands and grazed treescapes.

